# Access to healthcare for people with sickle cell disease: Views of healthcare professionals on policies and practices

**DOI:** 10.1002/mgg3.2142

**Published:** 2023-02-02

**Authors:** Obi Peter Adigwe

**Affiliations:** ^1^ National Institute for Pharmaceutical Research and Development Abuja Nigeria

**Keywords:** genotype, healthcare, Nigeria, policies, sickle cell

## Abstract

**Introduction:**

Sickle cell disease is a genetic disorder with its highest prevalence in Nigeria. The condition causes red blood cells to turn rigid, and consequently, results to several complications including organ damage. This study aimed at assessing views of health practitioners in Nigeria on policies and practices in the area of access to healthcare services for sickle cell disease.

**Methods:**

A cross‐sectional study was undertaken amongst healthcare professionals in Nigeria. Data were collected using a self‐administered questionnaire, and analyses were undertaken using Statistical Package for Social Sciences software.

**Results:**

A significant proportion of the participants (42.8%) disagreed that relevant legislative framework exists to facilitate optimal access to high‐quality healthcare services for persons with sickle cell disorder in Nigeria. Two‐thirds of the study cohort were of the opinion that public health surveillance towards sickle cell disease was suboptimal (61.2%). Also, more than three‐quarters of the respondents (78.7%) indicated that the cost of managing sickle cell disease was not affordable to majority of affected Nigerians.

**Conclusion:**

This study provides critical insights into access to healthcare services for sickle cell disease. As such, challenges preventing access to healthcare services for sickle cell patients which have been identified in this study can underpin the development of contextual policies to address them.

## 1 INTRODUCTION

Sickle cell disease is a genetic blood disorder that is characterised by haemolytic anaemia as well as recurrent vaso‐occlusive episodes which can result in painful crises, organ damage, morbidity and mortality (Platt et al., 1994; Powars et al., 2005; Smith et al., 2005). Approximately 25 million people are affected by the disease globally with majority of them living in sub‐Saharan Africa where Nigeria is reported to have the highest burden of the disease (Piel et al., 2013). Sickle cell disorder is the most common non‐infectious disease in this setting, with up to 25% of the population carrying sickle cell trait (Akodu et al., 2013). Many churches make it compulsory for intended couples to undertake haemoglobin electrophoresis tests and present the results before they are wedded. Whilst there have been several arguments challenging the constitutionality of this practice, this has however, not stopped these churches from carrying out this act (Yarhere et al., 2019). The reason attributed to this action is to reduce the prevalence of sickle cell disease due to the burden associated with it.

In most developed countries, new‐born screening programmes for sickle cell disease are routinely undertaken, which is due to the fact that early diagnosis can provide opportunities to quickly commence management that will consequently improve the quality of life of affected individuals (Aygun & Odame, 2012). However, in most parts of sub‐Saharan Africa, a lack of facilities and equipment prevent widespread access to similar interventions (Odunvbun et al., 2008). Additionally, there is evidence that suggests that in Africa, sickle cell disorder is associated with significant stigmatisation of both affected children and their families (Assimadi et al., 2000; Bamisaiye et al., 1974). Screening for the condition has been reported to cause disharmony, domestic violence, and breakdown of marriages due to stigmatisation (Marsh et al., 2011). Implementation of new‐born screening programmes in such communities, therefore, requires adequate enlightenment regarding the disease.

Addressing public health needs associated with sickle cell disease is an important strategy towards improving outcomes as well as for maintaining good quality of health for those affected by the disorder (Yusuf et al., 2011). As at 2018, Nigeria was ranked 187 of 191 countries in an assessment of the level of compliance with Universal Health Coverage (UHC), as only a small proportion of the population are health insured (Abubakar et al., 2018). The government provision for health is inadequate, and out‐of‐pocket payments for healthcare services causes households to incur significant amount of expenditure, which a considerable proportion of the populace is unable to afford (Aregbeshola & Khan, 2018).

Healthcare professionals are important stakeholders with first‐hand experience of barriers faced by patients as it relates to effective care (Houwing et al., 2021). Considering the burden of sickle cell disease in the Nigerian setting, it is of critical importance to explore the views and experiences of this category of stakeholders. This approach guarantees that government policymaking and professional practice reforms are underpinned by evidence‐based research. So far, no study has adopted this approach with respect to healthcare professionals' perspectives in the Nigerian setting. This study, therefore, aimed at exploring views of healthcare professionals in the country on policies and practices in the area of access to healthcare services for sickle cell disease.

## 2 METHODS

### 2.1 Ethical compliance

Ethical approval was obtained from the Federal Capital Territory Health Research Ethics Committee. The study was conducted in accordance with the declaration of Helsinki. Participation in the study was voluntary as written informed consents were obtained from participants prior to the administration of questionnaires. Confidentiality was maintained by not including the names of the study participants in the data collection tool.

### 2.2 Study tool design

A cross‐sectional design was adopted for the study and was undertaken in Nigeria between October and November 2021. A data collection tool was developed in the English language following an extensive literature review (Houwing et al., 2021; Kayle et al., 2020; Matthie et al., 2016). An iterative process involving a panel of faculty members in health sciences was used to develop the questionnaire items. The panel comprised four members who were involved in teaching and research activities in the area of sickle cell disease. A draft version of all the items in the study tool was reviewed by the panel; each person reviewed the items independently and suggested changes, additions and deletions. The revision process continued until a consensus was reached. The questionnaire items were structured to gain insights into policies and practices with respect to access to healthcare for sickle cell disease patients.

### 2.3 Questionnaire validation

Face and content validations of the instrument were undertaken using an independent expert panel comprising five members. The questionnaire items were assessed for appropriateness, complexity, attractiveness and relevance. Some of the statements were edited and reworded, whilst content validity was undertaken by quantitative method. Content validity ratio and content validity index were tested for each item, and only those that passed these tests were included in the final instrument. The questionnaire was pretested by administering it to initial cohort of 20 participants comprising randomly selected healthcare professionals from different disciplines. The feedback received did not necessitate any major change.

### 2.4 Data collection

The participants were selected using stratified multistage sampling method. Firstly, one state was selected randomly from six different geopolitical zones in Nigeria. Four health facilities were randomly selected from each state. In each facility, a number of participants were recruited using convenience sampling technique. Inclusion criteria were as follows: healthcare professionals who are practicing in Nigeria, being willing to participate in the study and working in a hospital setting. Participants who did not meet these criteria were excluded from the study. Paper‐based questionnaires were administered to doctors, pharmacists, medical laboratory scientists, nurses and other healthcare workers in several healthcare facilities visited.

### 2.5 Data analysis

Following the importation of data collected into Statistical Package for Social Sciences software version 25, descriptive statistical analysis was carried out. The association between variables was tested using chi‐square test. A *p*‐value of 0.05 or less was considered the threshold for statistical significance.

## 3 RESULTS

### 3.1 Demography

A total of 1200 questionnaires were administered and 1002 were completed and returned, giving a response rate of 83.5%. Male and female participants were of a similar proportion as indicated by 51.2% and 48.8% respectively. Close to a third of the participants (28.4%) were between 31 and 40 years. Other relevant details about socio‐demographic characteristics are presented in Table 1.

**TABLE 1:** Socio‐demographic characteristics

Variable	Frequency (%)
Gender	
Male	513 (51.2)
Female	489 (48.8)
Age (years)	
≤30	564 (56.3)
31–40	285 (28.4)
41–50	118 (11.8)
51 and above	17 (1.7)
Missing	18 (1.8)
Highest educational level	
Diploma	284 (28.3)
First degree	601 (60.0)
Masters' degree	88 (8.8)
PhD	8 (0.8)
Missing	21 (2.1)
Genotype	
I do not know	68 (6.8)
AA	652 (65.1)
AS	212 (21.2)
AC	33 (3.3)
SS	6 (0.6)
SC	2 (0.2)
Missing	29 (2.9)

PhD = Doctor of Philosophy.

### 3.2 Policy framework for sickle cell disease

**FIGURE 1:**
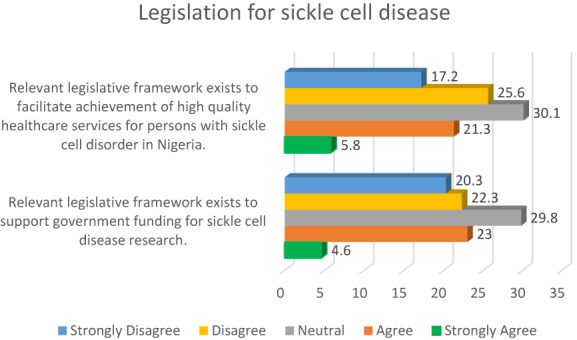
Legislative framework for sickle cell disease.

A significant proportion of the participants (42.8%) disagreed that relevant legislative framework exists to support high‐quality healthcare services for persons with sickle cell in Nigeria, whilst a third of the respondents (30.1%) were neutral on this issue. Other relevant details are presented in Figure 1.

Also, from Figure 1, a considerable proportion of the participants (42.6%) disagreed that relevant legislative framework exists to support government funding for sickle cell disease research.

### 3.3 Equipment and treatment facilities

**FIGURE 2:**
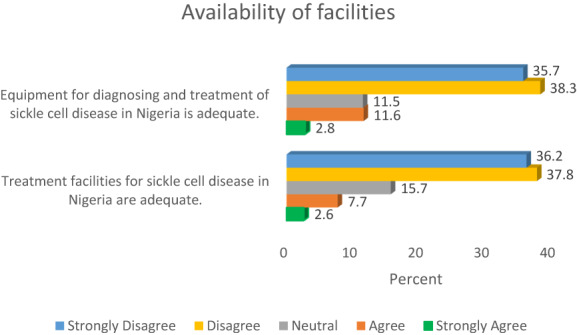
Availability of equipment and treatment facilities.

As presented in Figure 2, three‐quarters of the study cohort (74%) disagreed that equipment for diagnosing and managing sickle cell disease in Nigeria was adequate, whilst only 14% of the participants indicated adequacy of equipment needs with respect to diagnosis and treatment of the disease.

Also, from Figure 2, majority of the study participants (74%) disagreed that treatment facilities for sickle cell disease in Nigeria were adequate, whilst 15.7% were neutral in their responses.

### 3.4 Public health surveillance and sensitisation practices

**FIGURE 3:**
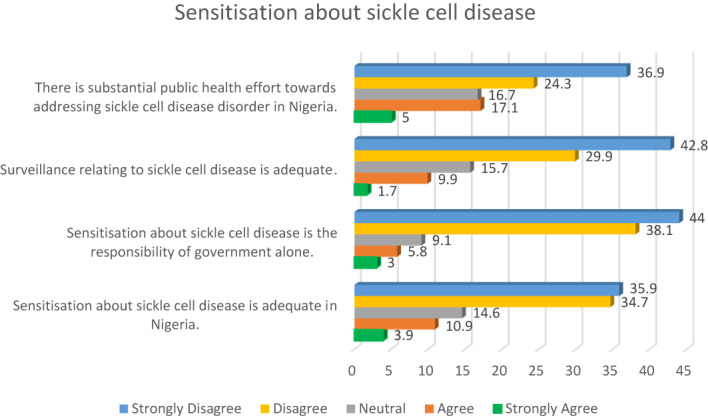
Public health surveillance and sensitisation towards sickle cell disease.

Whilst 22.1% of the participants indicated that substantial public health effort towards addressing sickle cell disease exists, two‐thirds of the respondents (61.2%) disagreed with this standpoint. Similarly, close to three‐quarters of the study participants (72.7%) disagreed that surveillance relating to sickle cell disease was adequate. Details are presented in Figure 3.

Furthermore, as presented in Figure 3, majority of the participants (70.6%) disagreed that sensitisation about sickle cell disease was adequate in Nigeria, whilst a strong majority of the respondents (82.1%) indicated that sensitisation about sickle cell disease was not the responsibility of government alone.

### 3.5 Sickle cell disease management practices

**FIGURE 4:**
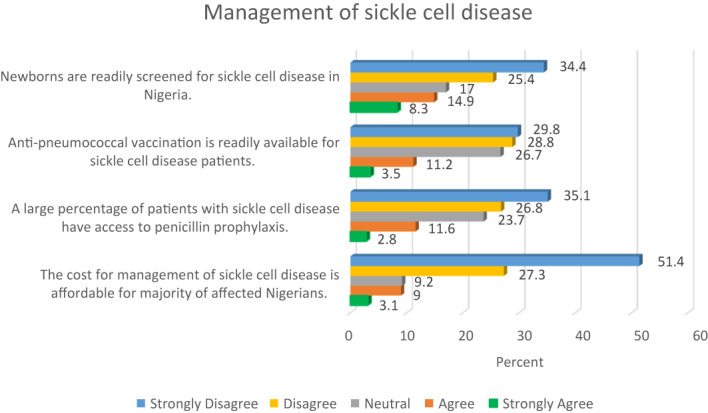
Sickle cell disease management.

More than half of the study participants (59.8%) disagreed that new‐borns were readily screened for sickle cell disease, and a similar proportion (58.6%) indicated that anti‐pneumococcal vaccination was not readily available for those with sickle cell disorder. Further details are presented in Figure 4.

Also, findings presented in Figure 4 show that about two‐thirds of the study cohort (61.9%) indicated that a large percentage of patients with sickle cell disease lacked access to penicillin prophylaxis, whilst more than three‐quarters of the participants (78.7%) were of the view that the cost of managing sickle cell disease was not affordable for majority of affected individuals.

Chi‐square test was further undertaken to determine association between demographic data and other variables. Findings indicate that a large proportion of those with diplomas (76.7%), first degrees (90.4%) and master's degrees (93.9%) disagreed that the cost of management of sickle cell disease was affordable to majority of Nigerians as compared to only 62.5% of those with doctorate qualifications who disagreed with this statement. This finding was statistically significant (*p* < 0.001). However, no significant relationship was observed between male and female participants with respect to affordability of the cost of managing sickle cell disease (*p* = 0.807). Few of the participants who are ≤30 years (16.4%), those between 31 and 40 years (10.7%), and those between 41 and 50 years of age (6.9%) were of the opinion that surveillance relating to sickle cell disease was adequate as compared to a quarter of respondents (26.7%) who are 51 years and above. This finding was also statistically significant (*p* = 0.013).

## 4 DISCUSSION

In this study, it emerged that a considerable proportion of the participants were of the view that there is a lack of relevant policy framework to achieve high‐quality care as well as support funding for sickle cell disease research. These findings suggest the urgent need for strengthening of relevant policies and legislation in this area so as to improve access to healthcare services for persons suffering from sickle cell disease; this is particularly important, considering the burden of sickle cell disease in Nigeria (Brown et al., 2010). Developing and implementing relevant policies to encourage funding and support for sickle cell disorder can go a long way in ameliorating the challenges associated with the disease in the Nigerian setting.

Findings from this study show that the respondents were of the opinion that equipment for sickle cell disease treatment as well as facilities for the management of the condition were inadequate and this is similar to previous findings in Nigeria (Galadanci et al., 2014). However, whilst this present study focuses on all healthcare professionals irrespective of their practice settings, the participants of the study reported by Galadanci et al. (2014) comprised only medical doctors in sickle cell–dedicated centres.

Participants in this study were of the opinion that public health efforts towards prevention of sickle cell disease as well as surveillance relating to the condition were inadequate. This novel finding may be responsible for suboptimal practices that may hamper mitigation strategies. An earlier study has reported willingness to marry amongst individuals with genotype incompatibility as well as disregards for genotype screening prior to relationships (Adigwe et al., 2022). What this implies, is an urgent need for government and stakeholders to intensify efforts in public awareness campaigns towards prevention and control of sickle cell disease so as to reduce the prevalence in Nigerian setting. This is especially important, given that between 2% and 3% of the population are affected with this disorder (Nwogoh et al., 2012; Serjeant & Serjeant, 2001), and about a quarter of the population are carriers of sickle cell trait (Akodu et al., 2013). Although participants were of the view that sensitisation about sickle cell disease was inadequate, they disagreed that the responsibility of sensitising the public about this disorder, be left to the government alone. This reinforces the need for a strategic multidisciplinary reforms that involve all relevant stakeholders in sickle cell prevention campaigns. Compared to their younger colleagues, significantly, more of the older participants (51 years and above) were of the opinion that surveillance relating to sickle cell disease was adequate. The reasons behind these demographic disparities are unclear, neccesitating the need further studies.

Majority of the participants indicated a lack of new‐born screening programmes for sickle cell disease in the study setting. This finding is particularly worrisome, given the evidence in the extant literature on the utility of new‐born screening for early diagnosis and management of the condition. This practice has been identified as a major factor responsible for the dramatic upturn in quality of life over the last few decades in USA (Quinn et al., 2010; Wang et al., 2011). Furthermore, participants indicated that a large proportion of sicle cell patients were challenged by a lack of anti‐pneumococcal vaccination as well as a lack of access to penicillin prophylaxis. These findings are contrary to best practices in more developed settings where improved access to penicillin prophylaxis and pneumococcal vaccination have helped to increase the life expectancy of persons affected with sickle cell disease (Cober & Phelps, 2010; Ellison et al., 2012; Hardie et al., 2009).

Findings from the study highlighted the high cost of managing sickle cell disease alongside the lack of affordability for majority of Nigerians, suggesting the need for a robust and contextual policy reforms that can alleviate the cost, whilst improving access. Intervention in this area is particularly important, considering the fact that majority of the populace access healthcare services in Nigeria through out‐of‐pocket payment (Abubakar et al., 2018; Aregbeshola & Khan, 2018). In other climes, government retains responsible for all, or atleast a substantial proportion of the cost of treatment relating to sickle cell disease. This strategy has contributed to improved quality of life for those affected with the condition (Davis et al., 1997; Pizzo et al., 2015).

This study aimed at exploring professional views through a cross‐sectional quantitative survey. Although the design aimed at a robust and comprehensive exploration, apotential limitation could arise due to the narrow methodological paradigm. As this is the first study to assess the views of different healthcare practitioners on access to healthcare services for sickle cell disease, adopting a mixed‐methods strategy that include a qualitative approach can provide deeper insights in further relevant studies.

## 5 CONCLUSION

Findings from this study revealed several novel sector‐wide observations that emerged from the unique perspective of healthcare professionals in Nigeria, such as inadequacies relating to healthcare services for sickle cell disease. Participants were of the opinion that relevant policy frameworks which facilitate attainment of high‐quality healthcare services for patients with sickle cell disease, were sub‐optimal.

Emergent evidence from the study indicated that facilities and equipment for the management of sickle cell disease were not adequate in the Nigerian healthcare setting. Also, public health efforts towards reducing the burden of the disease were reported as inadequate, which was worrisome, given the criticality of the public health interventions in prevention, control and management of the disease.

Participants in this study were of the opinion that public sensitisation about sickle cell disease was not adequate, even though this was identified as a critical tool in the prevention and control of the disease. It also emerged that such responsibility not be left to the government alone as non‐governmental stakeholders were exhorted to contribute more to advocacy and engagement activities that can underpin more effecient prevention and control of the disease.

Strategic use of the emergent findings of this study can give more fillip to the efforts aimed at reducing the high burden of sickle cell disease in Nigeria. Adoption of the study outcomes will enable the articulation and subsequent implementation of policy and practice refoms that facilitate contextual access to healthcare services for the condition. It is also of critical importance to emphasise the relevant contextual collaboration amongst all indicated stakeholders.

## 6 AUTHOR CONTRIBUTION

Obi Peter Adigwe is the sole author of this study.

## 7 ACKNWOLEDGEMENTS

None.

## 8 ETHICS STATEMENT

This study was approved by the Federal Capital Territory Health Research Ethics Committee (Approval number, FHREC/2021/01/97/12‐08‐21).
